# Supplementary material for: Difficulties and Psychological Impact of the SARS-CoV-2 Pandemic in Patients with Systemic Lupus Erythematosus: A Nationwide Patient Association Study
Source: Healthcare (Basel). 2022 Feb 9;10(2):330. doi: 10.3390/healthcare10020330 (PMC8872495; doi:10.3390/healthcare10020330)
Supplement: Supplementary file 1 [file healthcare-10-00330-s001.zip › healthcare-1565944-supplementary.pdf]

healthcare MDPI

Supplementary Table S1: Factors associated with the development of anxiety, depression or PTSD in univariate analysis (logistic regression).

	Odds ratio (95%CI) for anxiety	Odds ratio (95%CI) for depression	Odds ratio (95%CI) for PTSD
Difficulties to obtain HCQ	2.02 (1.17–3.50) $p = 0.01$	1.62 (0.95–2.77) $p = 0.07$	2.26 (1.30–3.92) $p = 0.004$
Difficulties to access medical care	1.97 (1.20–3.25) $p = 0.008$	2.64 (1.60–4.38) $p < 0.001$	2.16 (1.30–3.60) $p = 0.003$
Financial difficulties	1.41 (0.74–2.69) $p = 0.30$	2.62 (1.35–5.10), $p = 0.005$	2.57 (1.30–5.08) $p = 0.007$
Female Sex	1.99 (0.91–4.37) $p = 0.085$	1.31 (0.60–2.82) $p = 0.49$	3.30 (1.22–8.88) $P = 0.018$
Lupus flare	1.59 (1.00–2.52) $p = 0.05$	1.64 (1.03–2.60) $p = 0.04$	1.66 (1.03–2.66) $p = 0.04$
Reported COVID-19 disease	0.96 (0.50–1.82) $p = 0.89$	0.73 (0.37–1.42) $p = 0.35$	1.03 (0.53–1.98) $p = 0.93$
Use of teleconsultation	1.04 (0.68–1.61) $p = 0.85$	0.94 (0.6–1.45) $p = 0.77$	0.82 (0.52–1.28) $p = 0.38$
